# Diaqua­tetra­chloridotin(IV)–diglyme (1/2)

**DOI:** 10.1107/S1600536808029917

**Published:** 2008-09-20

**Authors:** James L. Wardell, William T. A. Harrison

**Affiliations:** a Departamento de Quimica, Universidade Federal de Minas Gerais, UFMG, Avenida Antônio Carlos 6627, Belo Horizonte, MG, CEP 31270-901, Brazil; b Department of Chemistry, University of Aberdeen, Meston Walk, Aberdeen AB24 3UE, Scotland

## Abstract

In the title 1:2 adduct, [SnCl_4_(H_2_O)_2_]·2C_6_H_14_O_3_, the Sn^IV^ atom (site symmetry 2) adopts a *cis*-SnO_2_Cl_4_ octa­hedral geometry. In the crystal structure, O—H⋯O hydrogen bonds lead to associations of one metal complex and two diglyme mol­ecules.

## Related literature

For related structures, see: Valle *et al.* (1984 ▶); Hough *et al.* (1986 ▶); Aza­dmehr *et al.* (2001 ▶). For further synthetic details, see: Hutton & Oakes (1976 ▶). For reference structural data, see: Allen *et al.* (1987 ▶). For bond valence sum calculations, see: Brese & O’Keeffe (1991 ▶).
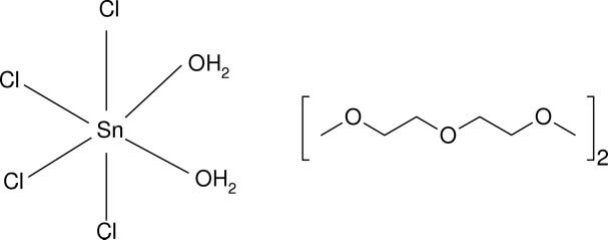


## Experimental

### 

#### Crystal data

[SnCl_4_(H_2_O)_2_]·2C_6_H_14_O_3_
*M* *_r_* = 564.86
Orthorhombic, 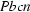

*a* = 8.4023 (2) Å
*b* = 17.1528 (3) Å
*c* = 15.9612 (4) Å
*V* = 2300.38 (9) Å^3^
*Z* = 4
Mo *K*α radiation
μ = 1.61 mm^−1^
*T* = 120 (2) K
0.55 × 0.43 × 0.15 mm

#### Data collection

Nonius KappaCCD diffractometer
Absorption correction: multi-scan (*SADABS*; Bruker, 2003 ▶) *T*_min_ = 0.472, *T*_max_ = 0.795
20197 measured reflections
2643 independent reflections
2292 reflections with *I* > 2σ(*I*)
*R*_int_ = 0.037

#### Refinement

*R*[*F* ^2^ > 2σ(*F* ^2^)] = 0.022
*wR*(*F* ^2^) = 0.050
*S* = 1.04
2643 reflections
121 parameters
H atoms treated by a mixture of independent and constrained refinement
Δρ_max_ = 0.56 e Å^−3^
Δρ_min_ = −0.50 e Å^−3^

### 

Data collection: *COLLECT* (Nonius, 1998 ▶); cell refinement: *SCALEPACK* (Otwinowski & Minor, 1997 ▶); data reduction: *SCALEPACK*, and *DENZO* (Otwinowski & Minor, 1997 ▶) and *SORTAV* (Blessing, 1995 ▶); program(s) used to solve structure: *SHELXS97* (Sheldrick, 2008 ▶); program(s) used to refine structure: *SHELXL97* (Sheldrick, 2008 ▶); molecular graphics: *ORTEP-3* (Farrugia, 1997 ▶); software used to prepare material for publication: *SHELXL97*.

## Supplementary Material

Crystal structure: contains datablocks I, global. DOI: 10.1107/S1600536808029917/bt2792sup1.cif

Structure factors: contains datablocks I. DOI: 10.1107/S1600536808029917/bt2792Isup2.hkl

Additional supplementary materials:  crystallographic information; 3D view; checkCIF report

## Figures and Tables

**Table 1:** Selected bond lengths (Å)

Sn1—O1	2.1343 (13)
Sn1—Cl1	2.3772 (4)
Sn1—Cl2	2.3853 (5)

**Table 2:** Hydrogen-bond geometry (Å, °)

*D*—H⋯*A*	*D*—H	H⋯*A*	*D*⋯*A*	*D*—H⋯*A*
O1—H2⋯O2	0.77 (2)	1.88 (2)	2.6503 (18)	175 (2)
O1—H1⋯O4	0.82 (2)	1.91 (2)	2.7296 (18)	175 (2)

**Fig. 1.**
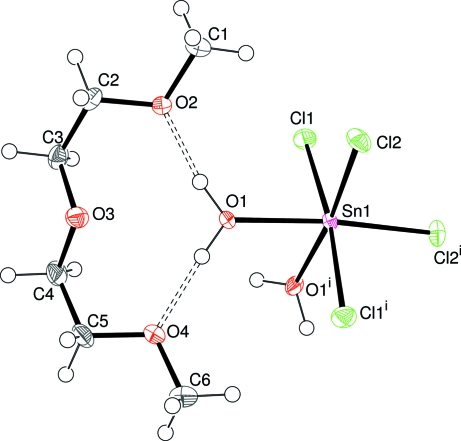
View of the molecular structure of (I) showing 50% displacement ellipsoids. The H atoms are drawn as spheres of arbitrary radius and the hydrogen bonds are shown as double-dashed lines. Symmetry code: (i) -x, y, 3/2 - z.

**Fig. 2.**
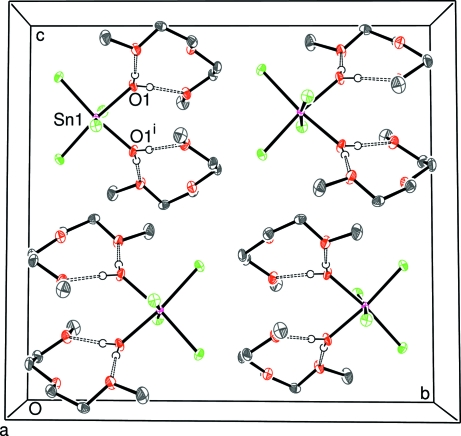
Unit cell packing for (I) showing the isolated hydrogen bonded assembiles of one metal complex and two diglyme molecules. Symmetry code: (i) -x, y, 3/2 - z. The C-bound H atoms are omitted for clarity.

## supplementary crystallographic information

### Comment

The title compound, (I), (Fig. 1) complements related adducts containing the same metal complex accompanied by various crown ethers (Valle *et al.*, 1984; Hough *et al.*, 1986; Azadmehr *et al.*, 2001).

In (I), the tin(IV) atom lies on a crystallographic twofold rotation axis, and bonds to two water molecules and four chloride ions, with the water O atoms in *cis* conformation {O1—Sn1—O1^i^ = 82.72 (8)°; i = -*x*, *y*, 3/2 - *z*]. Overall, a distorted octahedral coordination arises for the metal (Table 1). The bond valence sum (BVS) (Brese & O'Keeffe, 1991) for tin is 4.09 (expected value = 4.00).

In the crystal, the Sn(H_2_O)_2_Cl_4_ moiety links to two adjacent C_6_H_14_O_3_ (diglyme) molecules by way of O—H···O hydrogen bonds (Table 2), with each water molecule making two such bonds to the same diglyme species (Fig. 2). This hydrogen bonding pattern may correlate with the fact that the O—C—C—O torsion angles reflect *gauche* conformations about the C2—C3 and C4—C5 bonds [O2—C2—C3—O3 = 65.6 (2)°; O3—C4—C5—O4 = -64.9 (2)°], whereas the four C—C—O—C conformations are *trans*. Otherwise, the geometrical paramaters for (I) may be regarded as normal (Allen *et al.*, 1987).

### Experimental

Air-stable, colourless slabs of (I) were isolated from the slow evaporation of a methanolic solution (20 ml) containing 0.1 mmol C l_3_SnCH_2_CH_2_CO_2_H (Hutton & Oakes, 1976) and 0.1 mmol diglyme. M.P. 353–355 K. IR (KBr): 3500–2500, 1363, 1471, 1454, 1354,1287, 1250, 1141, 1102, 1079, 1105, 860, 834, 701, 617 cm^-1^ Anal: Calc: C 25.52; H 5.71%. Found: C 25.23; H 5.85%.

### Refinement

The water H atoms were located in a difference map and their positions were freely refined with the constraint *U*_iso_(H) = 1.2*U*_eq_(O). The C-bound H atoms were placed in calculated positions (C—H = 0.98–0.99 Å) and refined as riding with *U*_iso_(H) = 1.2*U*_eq_(C) or 1.5*U*_eq_(methyl C). The methyl groups were allowed to rotate, but not to tip, to best fit the electron density.

### Crystal data

**Table:**

[SnCl_4_(H_2_O)_2_]·2C_6_H_14_O_3_	*F*(000) = 1144
*M**_r_* = 564.86	*D*_x_ = 1.631 Mg m^−^^3^
Orthorhombic, *P**b**c**n*	Mo *K*α radiation, λ = 0.71073 Å
Hall symbol: -P 2n 2ab	Cell parameters from 10481 reflections
*a* = 8.4023 (2) Å	θ = 2.9–27.5°
*b* = 17.1528 (3) Å	µ = 1.61 mm^−^^1^
*c* = 15.9612 (4) Å	*T* = 120 K
*V* = 2300.38 (9) Å^3^	Slab, colourless
*Z* = 4	0.55 × 0.43 × 0.15 mm

### Data collection

**Table:**

Nonius KappaCCD diffractometer	2643 independent reflections
Radiation source: fine-focus sealed tube	2292 reflections with *I* > 2σ(*I*)
graphite	*R*_int_ = 0.037
ω and φ scans	θ_max_ = 27.6°, θ_min_ = 3.5°
Absorption correction: multi-scan (SADABS; Bruker, 2003)	*h* = −10→10
*T*_min_ = 0.472, *T*_max_ = 0.795	*k* = −19→22
20197 measured reflections	*l* = −17→20

### Refinement

**Table:**

Refinement on *F*^2^	Secondary atom site location: difference Fourier map
Least-squares matrix: full	Hydrogen site location: difmap and geom
*R*[*F*^2^ > 2σ(*F*^2^)] = 0.022	H atoms treated by a mixture of independent and constrained refinement
*wR*(*F*^2^) = 0.050	*w* = 1/[σ^2^(*F*_o_^2^) + (0.0206*P*)^2^ + 1.2513*P*] where *P* = (*F*_o_^2^ + 2*F*_c_^2^)/3
*S* = 1.04	(Δ/σ)_max_ < 0.001
2643 reflections	Δρ_max_ = 0.56 e Å^−^^3^
121 parameters	Δρ_min_ = −0.50 e Å^−^^3^
0 restraints	Extinction correction: (SHELXL97; Sheldrick, 2008), Fc^*^=kFc[1+0.001xFc^2^λ^3^/sin(2θ)]^-1/4^
Primary atom site location: structure-invariant direct methods	Extinction coefficient: 0.00095 (17)

### Special details

**Table:**

Geometry. All e.s.d.'s (except the e.s.d. in the dihedral angle between two l.s. planes) are estimated using the full covariance matrix. The cell e.s.d.'s are taken into account individually in the estimation of e.s.d.'s in distances, angles and torsion angles; correlations between e.s.d.'s in cell parameters are only used when they are defined by crystal symmetry. An approximate (isotropic) treatment of cell e.s.d.'s is used for estimating e.s.d.'s involving l.s. planes.
Refinement. Refinement of *F*^2^ against ALL reflections. The weighted *R*-factor *wR* and goodness of fit *S* are based on *F*^2^, conventional *R*-factors *R* are based on *F*, with *F* set to zero for negative *F*^2^. The threshold expression of *F*^2^ > σ(*F*^2^) is used only for calculating *R*-factors(gt) *etc*. and is not relevant to the choice of reflections for refinement. *R*-factors based on *F*^2^ are statistically about twice as large as those based on *F*, and *R*- factors based on ALL data will be even larger.

### Fractional atomic coordinates and isotropic or equivalent isotropic displacement parameters (Å^2^)

**Table:**

	*x*	*y*	*z*	*U*_iso_*/*U*_eq_	
Sn1	0.0000	0.165876 (9)	0.7500	0.01280 (7)	
Cl1	0.27717 (5)	0.17456 (3)	0.72161 (3)	0.02257 (11)	
Cl2	0.04156 (6)	0.07342 (3)	0.85946 (3)	0.02251 (11)	
O1	0.02883 (16)	0.25927 (8)	0.83704 (9)	0.0201 (3)	
H1	−0.023 (2)	0.2999 (14)	0.8351 (14)	0.024*	
H2	0.107 (3)	0.2655 (12)	0.8617 (14)	0.024*	
C1	0.3671 (3)	0.19896 (12)	0.94703 (16)	0.0380 (6)	
H1A	0.3444	0.1587	0.9051	0.057*	
H1B	0.3199	0.1839	1.0008	0.057*	
H1C	0.4826	0.2045	0.9535	0.057*	
C2	0.3303 (2)	0.33246 (10)	0.97905 (12)	0.0211 (4)	
H2A	0.4454	0.3351	0.9920	0.025*	
H2B	0.2720	0.3220	1.0318	0.025*	
C3	0.2759 (2)	0.40787 (10)	0.94202 (13)	0.0228 (4)	
H3A	0.3058	0.4516	0.9793	0.027*	
H3B	0.3273	0.4161	0.8869	0.027*	
C4	0.0482 (2)	0.46917 (10)	0.88487 (13)	0.0254 (4)	
H4A	0.1021	0.4714	0.8298	0.030*	
H4B	0.0690	0.5187	0.9148	0.030*	
C5	−0.1273 (2)	0.45811 (11)	0.87301 (13)	0.0257 (4)	
H5A	−0.1798	0.4520	0.9281	0.031*	
H5B	−0.1733	0.5044	0.8449	0.031*	
C6	−0.3192 (2)	0.38022 (12)	0.80483 (15)	0.0327 (5)	
H6A	−0.3336	0.3337	0.7700	0.049*	
H6B	−0.3587	0.4261	0.7747	0.049*	
H6C	−0.3786	0.3740	0.8573	0.049*	
O2	0.30068 (16)	0.27173 (7)	0.92018 (9)	0.0260 (3)	
O3	0.10705 (14)	0.40525 (7)	0.93230 (8)	0.0210 (3)	
O4	−0.15401 (14)	0.39017 (7)	0.82313 (8)	0.0226 (3)	

### Atomic displacement parameters (Å^2^)

**Table:**

	*U*^11^	*U*^22^	*U*^33^	*U*^12^	*U*^13^	*U*^23^
Sn1	0.01400 (9)	0.00920 (10)	0.01519 (11)	0.000	−0.00062 (6)	0.000
Cl1	0.0149 (2)	0.0241 (2)	0.0287 (3)	0.00062 (16)	0.00141 (18)	−0.00129 (19)
Cl2	0.0303 (2)	0.0155 (2)	0.0217 (3)	0.00401 (17)	−0.00013 (18)	0.00507 (18)
O1	0.0210 (7)	0.0128 (6)	0.0265 (8)	0.0060 (5)	−0.0101 (6)	−0.0061 (6)
C1	0.0429 (13)	0.0233 (11)	0.0477 (15)	0.0116 (9)	−0.0239 (11)	−0.0025 (10)
C2	0.0193 (8)	0.0252 (10)	0.0187 (11)	−0.0034 (7)	−0.0042 (7)	−0.0030 (7)
C3	0.0215 (9)	0.0206 (9)	0.0263 (12)	−0.0066 (7)	−0.0017 (8)	−0.0031 (8)
C4	0.0341 (10)	0.0129 (9)	0.0291 (12)	−0.0003 (7)	−0.0054 (9)	0.0018 (8)
C5	0.0334 (11)	0.0165 (9)	0.0273 (12)	0.0086 (7)	−0.0029 (8)	−0.0030 (8)
C6	0.0214 (10)	0.0325 (11)	0.0441 (14)	0.0059 (8)	−0.0066 (9)	−0.0025 (10)
O2	0.0300 (7)	0.0179 (6)	0.0300 (8)	0.0067 (5)	−0.0164 (6)	−0.0047 (6)
O3	0.0199 (6)	0.0170 (6)	0.0260 (8)	−0.0009 (5)	−0.0016 (5)	0.0034 (5)
O4	0.0196 (6)	0.0183 (6)	0.0299 (8)	0.0052 (5)	−0.0034 (5)	−0.0035 (6)

### Geometric parameters (Å, °)

**Table:**

Sn1—O1	2.1343 (13)	C2—H2B	0.9900
Sn1—O1^i^	2.1343 (13)	C3—O3	1.428 (2)
Sn1—Cl1	2.3772 (4)	C3—H3A	0.9900
Sn1—Cl1^i^	2.3772 (4)	C3—H3B	0.9900
Sn1—Cl2^i^	2.3853 (5)	C4—O3	1.421 (2)
Sn1—Cl2	2.3853 (5)	C4—C5	1.499 (3)
O1—H1	0.82 (2)	C4—H4A	0.9900
O1—H2	0.77 (2)	C4—H4B	0.9900
C1—O2	1.433 (2)	C5—O4	1.429 (2)
C1—H1A	0.9800	C5—H5A	0.9900
C1—H1B	0.9800	C5—H5B	0.9900
C1—H1C	0.9800	C6—O4	1.429 (2)
C2—O2	1.425 (2)	C6—H6A	0.9800
C2—C3	1.494 (2)	C6—H6B	0.9800
C2—H2A	0.9900	C6—H6C	0.9800
			
O1—Sn1—O1^i^	82.72 (8)	H2A—C2—H2B	108.4
O1—Sn1—Cl1	88.04 (4)	O3—C3—C2	108.66 (14)
O1^i^—Sn1—Cl1	86.57 (4)	O3—C3—H3A	110.0
O1—Sn1—Cl1^i^	86.57 (4)	C2—C3—H3A	110.0
O1^i^—Sn1—Cl1^i^	88.04 (4)	O3—C3—H3B	110.0
Cl1—Sn1—Cl1^i^	172.81 (2)	C2—C3—H3B	110.0
O1—Sn1—Cl2^i^	172.96 (4)	H3A—C3—H3B	108.3
O1^i^—Sn1—Cl2^i^	90.32 (4)	O3—C4—C5	108.18 (15)
Cl1—Sn1—Cl2^i^	92.608 (16)	O3—C4—H4A	110.1
Cl1^i^—Sn1—Cl2^i^	92.169 (17)	C5—C4—H4A	110.1
O1—Sn1—Cl2	90.32 (4)	O3—C4—H4B	110.1
O1^i^—Sn1—Cl2	172.96 (4)	C5—C4—H4B	110.1
Cl1—Sn1—Cl2	92.169 (16)	H4A—C4—H4B	108.4
Cl1^i^—Sn1—Cl2	92.608 (16)	O4—C5—C4	109.14 (14)
Cl2^i^—Sn1—Cl2	96.65 (2)	O4—C5—H5A	109.9
Sn1—O1—H1	123.4 (16)	C4—C5—H5A	109.9
Sn1—O1—H2	122.1 (16)	O4—C5—H5B	109.9
H1—O1—H2	111 (2)	C4—C5—H5B	109.9
O2—C1—H1A	109.5	H5A—C5—H5B	108.3
O2—C1—H1B	109.5	O4—C6—H6A	109.5
H1A—C1—H1B	109.5	O4—C6—H6B	109.5
O2—C1—H1C	109.5	H6A—C6—H6B	109.5
H1A—C1—H1C	109.5	O4—C6—H6C	109.5
H1B—C1—H1C	109.5	H6A—C6—H6C	109.5
O2—C2—C3	108.58 (15)	H6B—C6—H6C	109.5
O2—C2—H2A	110.0	C2—O2—C1	111.81 (14)
C3—C2—H2A	110.0	C4—O3—C3	112.30 (13)
O2—C2—H2B	110.0	C6—O4—C5	111.33 (14)
C3—C2—H2B	110.0		
			
O2—C2—C3—O3	65.6 (2)	C5—C4—O3—C3	176.00 (15)
O3—C4—C5—O4	−64.9 (2)	C2—C3—O3—C4	−170.23 (16)
C3—C2—O2—C1	172.98 (17)	C4—C5—O4—C6	−175.75 (16)

Symmetry codes: (i) −*x*, *y*, −*z*+3/2.

### Hydrogen-bond geometry (Å, °)

**Table:**

*D*—H···*A*	*D*—H	H···*A*	*D*···*A*	*D*—H···*A*
O1—H2···O2	0.77 (2)	1.88 (2)	2.6503 (18)	175 (2)
O1—H1···O4	0.82 (2)	1.91 (2)	2.7296 (18)	175 (2)
